# A radical regulator: 14-3-3-mediated methionine oxidation of transcription factor BSR-D1 to counter blast fungus in rice

**DOI:** 10.1093/plcell/koag058

**Published:** 2026-03-05

**Authors:** Ved Prakash

**Affiliations:** Assistant Features Editor, The Plant Cell, American Society of Plant Biologists, United States; Department of Plant Pathology, The Ohio State University, Wooster, OH 44691, United States

Plant survival in a particular environment depends on an active surveillance and rapid response system. When a pathogen attacks, the plant begins a rapid counter-attack by inducing synthesis of various biochemicals. The induction of reactive oxygen species, such as hydrogen peroxide (H_2_O_2_), is one example, which is central to the defense against the blast fungus (Li et al. 2017). However, the plant must precisely control the amount of such chemicals; while high reactive oxygen species levels can stop a pathogen, long exposure to oxidative stress can damage a plant's own proteins and DNA. To balance defense and growth, plants have evolved redox switches (proteins) that sense the chemical state of the cell and trigger expression of specific genes to either increase defense or maintain physiological balance. It is important to understand these molecular switches in order to engineer crops that are resistant to diseases.

Recent work by Tianyu Zhou and colleagues (Zhou et al. 2026) unraveled a sophisticated signaling network in rice involving H_2_O_2_; the highly conserved 14-3-3 protein, OsGF14d (Cui et al. 2022); and BSR-D1, a transcription factor previously known as a negative regulator of rice blast resistance (Zhu et al. 2020) (Fig. 1). Using in vitro pull down assay and in vivo co-immunoprecipitation assay, the authors reported a direct interaction between OsGF14d and BSR-D1. This study also revealed a novel, non-enzymatic function of a 14-3-3 protein in which it facilitates oxidation of Met187 on BSR-D1. The authors used AlphaFold 3 modeling and electrostatic mapping to show that OsGF14d creates a specialized acidic microenvironment that is critical for lowering the chemical barrier for the oxidation of a specific methionine residue, Met187, on the BSR-D1 protein.

**Figure 1:**
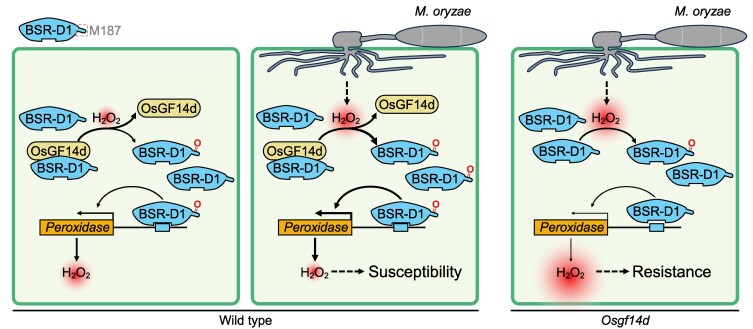
Proposed model for OsGF14d-BSR-D1–mediated disease resistance. In wild-type rice, OsGF14d enhances BSR-D1 oxidation, which increases the transcriptional activity of peroxidase gene and reduction of H_2_O_2_ level, leading to susceptibility. In Osgf14d knockout lines, BSR-D1 oxidation is lowered, ultimately leading to resistance. Reprinted from Zhou et al. (2026), Figure 7.

To confirm that this oxidation occurs within living plant cells, the researchers employed redox-activated chemical tagging. This method allowed them to observe that the surge of H_2_O_2_ caused by *M. oryzae* infection directly converts Met187 into methionine sulfoxide. This chemical modification (the addition of a single oxygen atom) functions as a powerful conformational “on-switch”: once oxidized, BSR-D1 increases its binding affinity for the promoters of target peroxidase genes. These peroxidases then break down H_2_O_2_. Thus, the oxidation of Met187 turns inactive BSR-D1 into a potent activator of plant's antioxidant defenses, effectively dampening the immune response.

The functional significance of this pathway was validated using *OsGF14d*-knockout (*OsGF14d*-KO) and overexpression (*OsGF14d*-OX) rice lines. Zhou et al. found that upon *M. oryzae* infection, *OsGF14d*-KO shows enhanced resistance (fewer lesions and 50% reduction in fungal biomass), while *OsGF14d*-OX exhibits increased susceptibility (more lesions and 3 times more fungal biomass) compared to wild type. In addition, BSR-D1 could not be efficiently oxidized and remained in its “off” state in *OsGF14d* knockout lines. This prevents the activation of the peroxidase, allowing H_2_O_2_ to remain at high levels that successfully control *M. oryzae* spread. Importantly, *OsGF14d* knockout lines showed no significant differences in seed setting rate, tillering number, grain number per spike, or grain weight compared to standard rice varieties. This indicates that disrupting this specific “off-switch” enhances disease resistance without the typical “growth-defense tradeoff” that often plagues crop engineering.

In conclusion, this work provides a unique mechanism where a 14-3-3 protein facilitates a site-specific Met oxidation to modulate plant immunity against the blast fungus. *OsGF14d* could be a promising target for genome editing for developing durable, broad-spectrum resistance in cereal crops. It would be interesting to explore whether the creation of acidic microenvironment is a universal strategy used by 14-3-3 proteins to regulate transcription factors under other biotic or abiotic stresses.

## Recent related articles in *The Plant Cell*:

- Yan et al. (2023) performed transcriptional profiling of rice blast infection, providing researchers a good resource to understand the transcriptional changes associated with the disease.
- Wang et al. (2024) reported that rice MYB transcription factor MYB110 negatively regulates plant height.
- Ma et al. (2023) elucidated the molecular mechanism of osmotic stress tolerance in rice and showed that the 14-3-3 protein OsGF14f is a positive regulator of osmotic stress tolerance.

## Data Availability

No new data were generated or analysed in support of this research.

## Dive Curated Terms

The following phenotypic, genotypic, and functional terms are of significance to the work described in this paper:

MYB110 Gramene: AT3G29020
MYB110 Araport: AT3G29020
